# Population Genetic Structure of Three‐Spined Sticklebacks in the St. Lawrence: A Gradient of Change

**DOI:** 10.1002/ece3.71153

**Published:** 2025-04-23

**Authors:** Sann Delaive, Florent Sylvestre, Amanda Xuereb, Laurie Lecomte, Brian Boyle, Christian Otis, Louis Bernatchez, Nicolas Derome

**Affiliations:** ^1^ Département de Biologie, Institut de Biologie Intégrative et Des Systèmes (IBIS) Université Laval Québec Quebec Canada; ^2^ Institut de Biologie Intégrative et Des Systèmes Université Laval Québec Quebec Canada

**Keywords:** *Gasterosteus aculeatus*, population structure, salinity gradient, whole‐genome sequencing

## Abstract

Understanding how environmental gradients shape population genetic structure is critical for elucidating evolutionary dynamics in heterogeneous landscapes. The St. Lawrence Estuary, spanning fluvial, middle, and marine zones, presents a steep salinity gradient that serves as an ideal setting to study such a question. Three‐spined sticklebacks ( *Gasterosteus aculeatus* ) thrive across these zones, offering an ideal model system to investigate the interplay of gene flow and natural selection in shaping population structure. Using whole‐genome resequencing of sticklebacks from 12 sites, this study aimed to resolve fine‐scale population structure and investigate how genetic diversity and differentiation are influenced by selection and gene flow. By integrating single nucleotide polymorphisms (SNPs) and structural variants (SVs), we assessed differentiation patterns, examined clinal variation, and evaluated the relative roles of gene flow and selection in shaping population dynamics. Our findings reveal clear genetic differentiation between fluvial and saltwater populations, with Baie‐Saint‐Paul forming a potential third group. Salinity emerged as a key driver of genetic structure, with clinal variation in allele frequencies suggesting ongoing adaptation along the gradient. Demographic modeling indicated a history of secondary contact with recent and weak gene flow. Structural variants, particularly indels, complemented SNP‐based analyses, underscoring their importance in detecting fine‐scale population structure. These results highlight the complex interplay of evolutionary forces shaping biodiversity in transitional environments, providing a basis for exploring local adaptation in connected populations and contributing to broader efforts in conservation genomics.

## Graphical abstract

This study investigates how environmental gradients shape genetic population structure using three‐spined sticklebacks across the St. Lawrence Estuary's salinity gradient. Whole‐genome analyses reveal clear differentiation between fluvial and saltwater populations, with evidence of a third group at Baie‐Saint‐Paul. Salinity‐driven clinal variation, secondary contact with weak gene flow, and the importance of structural variants highlight the complex interplay of selection and gene flow in shaping biodiversity in transitional environments.

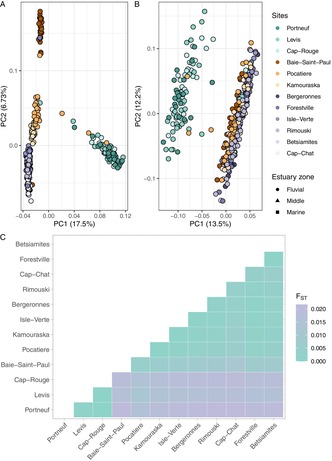

## 1 Introduction

Understanding how evolutionary forces shape the spatial distribution of genetic diversity within a species is essential for characterizing its evolutionary history, understanding the distribution and connectivity of its populations, and designing and implementing conservation efforts (Waples et al. 2008; Ouborg 2009). Natural selection and genetic drift drive differentiation between populations, while gene flow acts as a homogenizing force that reduces the differentiation between populations (Cowen and Sponaugle 2009). In marine environments, clear physical boundaries for dispersal are often absent, leading to considerable levels of gene flow. Additionally, marine species typically have large population sizes, making them less vulnerable to genetic drift (Bachmann et al. 2020). An important level of gene flow combined with reduced genetic drift results in relatively weak signals of population differentiation (Waples 1998) that may be driven by geographically limited dispersal patterns such as isolation by distance (IBD) (Perez et al. 2018), or by differences in environmental conditions (Bernatchez 2016).

Three‐spined sticklebacks ( *Gasterosteus aculeatus* , Linnaeus 1758) thrive in both freshwater and marine environments, with freshwater populations originating from recurrent colonization events by the ancestral marine ecotype following glacial cycles approximately 10 million years ago (Mäkinen et al. 2008). Due to repetitive colonization, three‐spined sticklebacks have garnered recognition as a model species for the study of parallel evolution, adaptive radiation, and speciation (Ostlund‐Nilsson et al. 2006). This status has facilitated the availability of valuable resources such as a refined reference genome, an annotated transcriptome, and a comprehensive genetic map (Reid et al. 2021).

Additionally, numerous studies have delved into the adaptation of three‐spined stickleback to freshwater environments, accumulating a large body of information on both phenotypic and genetic differences between the two ecotypes (Colosimo et al. 2004; Chan et al. 2010; Hohenlohe et al. 2010; Jones et al. 2012). Notably, the presence of standing genetic variation in the ancestral marine population has been established as a key factor enabling the rapid and repeated adaptation of sticklebacks to freshwater. Many freshwater‐adaptive alleles predate colonization events and persist at low frequencies in marine populations, allowing for swift evolutionary responses when new freshwater habitats become available. This reservoir of genetic variation has played a crucial role in shaping parallel adaptation across geographically distinct freshwater populations (Jones et al. 2012; Fang et al. 2020; Roberts Kingman et al. 2021).

Much of this work has focused on the differentiation between two main migratory forms: freshwater resident populations, which remain in freshwater year‐round, and anadromous populations, which are considered as the ancestral form and migrate to freshwater or estuaries to reproduce in spring. These forms often exhibit reproductive isolation, as demonstrated in populations from Japan and Ireland (Kitano et al. 2012; Dean et al. 2019), and show distinct morphological traits, such as differences in lateral plate number and pelvic spine length (Bell 2001; Colosimo et al. 2004), as well as physiological and behavioral traits (Tudorache et al. 2007; Seebacher et al. 2016; Barnes et al. 2024). However, little is known about how anadromous populations are spatially structured or the extent to which adaptive differentiation can occur between connected populations, particularly along continuous and heterogeneous environmental gradients (McCairns and Bernatchez 2008; Guo et al. 2015; Bal et al. 2021).

**FIGURE 1:**
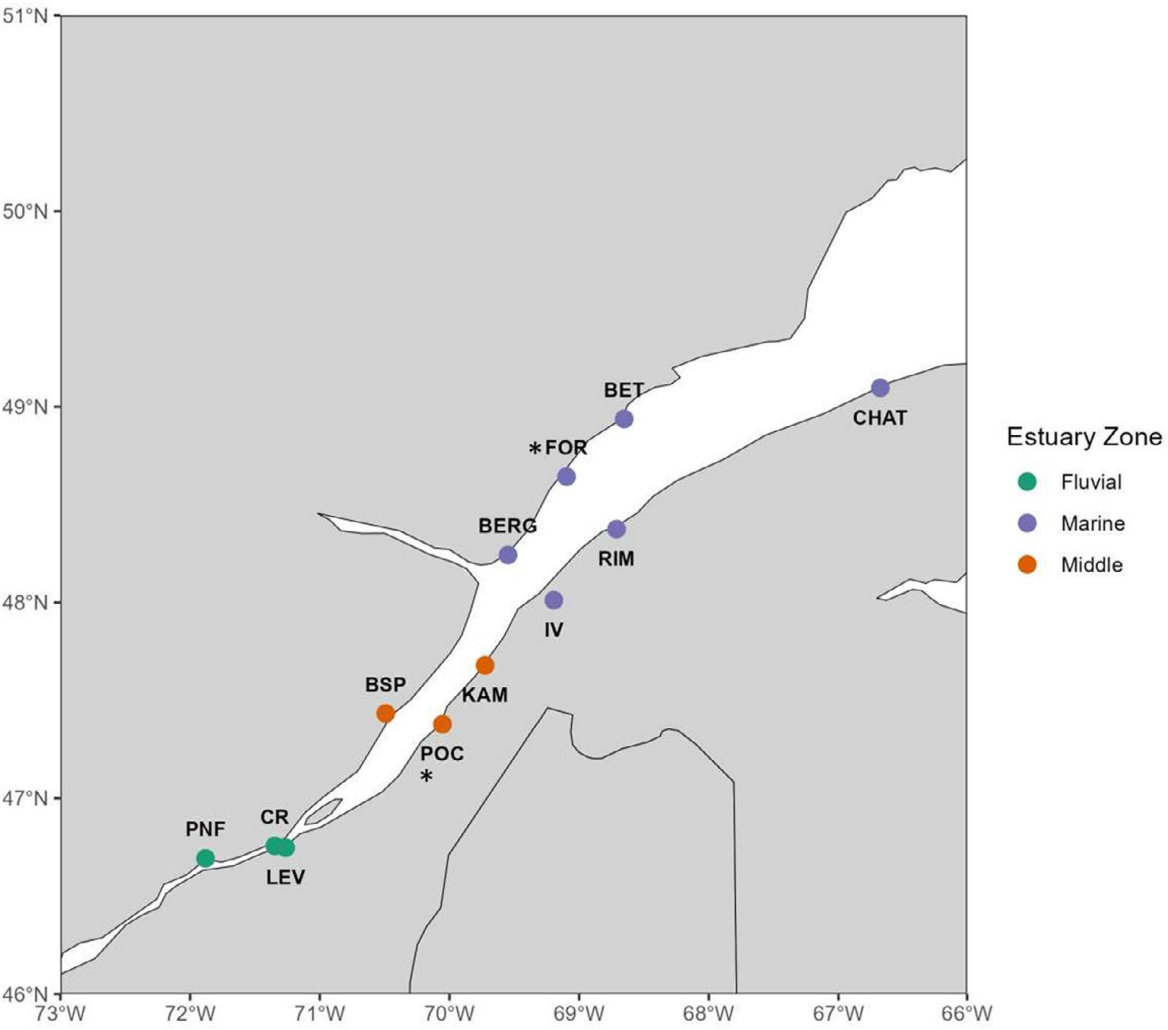
Sampling sites of three‐spined sticklebacks across environmental gradients of the St. Lawrence Estuary (2021). The * represents sites that were not present in McCairns and Bernatchez's (2010) study.

The St. Lawrence Estuary is divided into three distinct zones, each of which manifests unique environmental characteristics and is inhabited by three‐spined sticklebacks: the fluvial, middle, and marine estuary (Figure 1). The fluvial estuary is characterized by freshwater conditions and experiences elevated temperatures during the summer. In contrast, the marine estuary is marked by marine conditions, featuring a salinity around 30 ppm and colder water temperatures. The middle estuary encompasses a broad spectrum of environmental conditions, ranging from salinity of 5 to 30 ppm (Dolgopolova and Isupova 2011). Recent colonization scenarios suggest that three‐spined sticklebacks in the Estuary came from a Western Atlantic ancestral population (Haines 2023). These sticklebacks are generally considered anadromous; however, little is known about their migration routes and their overwintering locations. Unlike most estuarine populations, St. Lawrence sticklebacks show no variation in lateral plate number (McCairns and Bernatchez 2012) and are fully plated in both the fluvial and marine estuary. Despite this morphological similarity, sticklebacks from the fluvial estuary exhibit distinct reproductive behaviors compared to those from the middle and marine estuary. In the fluvial estuary, reproduction occurs in calm nearshore waters or at river mouths, whereas in the middle and marine estuary, sticklebacks spawn in small ponds within salt marshes, accessible only during high spring tides. Common garden experiments have shown that fluvial and marine estuary sticklebacks can interbreed without any detectable impact on offspring viability (McCairns and Bernatchez 2010). Previous studies of population structure and adaptive dynamics in anadromous three‐spined sticklebacks in the St. Lawrence estuary using 10 microsatellites identified genetic differentiation between fluvial sticklebacks and those inhabiting the marine and middle estuary (McCairns and Bernatchez 2008). Given the relatively low levels of genetic differentiation and the capacity for interbreeding, these studies suggested ongoing gene flow among populations across Estuary zones.

In this study, our objective was to uncover the fine‐scale population structure of an anadromous form of three‐spined stickleback across the continuous and heterogeneous environment of the St. Lawrence Estuary using a more comprehensive genomic dataset compared to previous studies that were based primarily on microsatellite markers, which are limited in power to detect subtle differentiation (Ryman et al. 2006; DeFaveri et al. 2013). Here, we leverage a genome‐wide SNP dataset, which offers higher‐resolution insights (Benestan et al. 2015; Xuereb et al. 2022; Pinsky et al. 2023), alongside SVs, which represents another important type of genetic variation. While both SNPs and SVs contribute to resolving population structure, SVs capture large‐scale genetic changes that may not be detected by SNPs alone, providing a complementary perspective on genomic differentiation (Dorant et al. 2020; Mérot et al. 2020; Weissensteiner et al. 2020; Lecomte et al. 2024). We hypothesized that a finer population structure exists within the middle estuary and that IBD alone would not sufficiently explain the population differentiation between estuary zones. In the presence of both gene flow and selection by environmental conditions, we expected to find genomic signatures of differentiation, characterized by distinct patterns of genetic diversity and clinal variation in allele frequencies, reflecting the interplay of gene flow and selection as evolutionary forces shaping population structure along the St. Lawrence Estuary. While SNPs and SVs can play independent roles in adaptation, we focus on examining population structure and demographic history along an environmental gradient rather than on characterizing the genomic basis of adaptation. This study provides a foundation for in‐depth investigation into the biological function and frequency patterns of SVs across Estuary zones and the relative contribution of SNPs and SVs to fine‐scale adaptation of three‐spined stickleback in this region.

## 2 Methods

### 2.1 Ethics Statement

This study was approved by the Comité de Protection des Animaux de l'Université Laval (CPAUL, approval number SIRUL 053918) and by the Ministère des Forêts, de la Faune et des Parcs du Québec (permit number 2020041500400SP) for fish sampling.

### 2.2 Sampling

In the summer of 2021, we sampled 460 three‐spined sticklebacks across 12 sites situated within the St. Lawrence Estuary (Figure 1). To ensure a representative coverage of environmental gradients characterizing the Estuary, we selected three sites within the fluvial estuary, three within the medium estuary (middle), and six within the marine estuary. Field observations and information about sampling sites from McCairns and Bernatchez (2008) were used to identify breeding ponds along the Estuary.

Within the fluvial estuary, two sites (Lévis and Portneuf) were sampled using a wide fishnet deployed directly in the St. Lawrence River. The third fluvial site (Cap‐Rouge) presented accessibility challenges and was sampled using minnow traps. In the medium and marine estuary, we targeted stickleback breeding sites consisting of ponds situated within the tidal zone. Nine sampling sites were identified, comprising three within the medium estuary (Baie‐Saint‐Paul, La Pocatière, and Kamouraska), two at the interface of the marine and the medium estuary (Isle‐Verte and Bergeronnes), and four within the marine estuary (Betsiamites, Forestville, Rimouski, and Cap‐Chat).

At each sampling site, we collected 40 individuals with a balanced sex ratio. Following capture, individuals were euthanized on‐site using MS222. Subsequently, we sampled fin clips for each specimen. Fin clips were preserved in 95% ethanol, while bodies were initially stored on ice before being transferred to a −20°C freezer for long‐term preservation.

### 2.3 DNA Extraction, Library Preparation and Genome Sequencing

We extracted genomic DNA from the fin tissue using a well‐established salt‐based protocol modified by Aljanabi and Martinez (1997). We assessed DNA quality by migration on an agarose gel and its purity using a nanodrop spectrophotometer.

Following quality control procedures, all samples were normalized to 1 μL/mL. Individual libraries were constructed following a modified version of the Nextera protocol (Therkildsen and Palumbi 2017; Mérot et al. 2021). A total of five libraries were prepared, each consisting of 96 samples, with eight samples per site and an equilibrated sex ratio. The libraries were pooled to equimolarity and checked for quality using a DNA chip. Sequencing was performed at Génome Québec Innovation Centre (Montréal, QC) on the Illumina NovaSeq 6000 S4 platform with paired‐end sequencing (PE150) targeting a coverage of 15× across the whole genome.

To construct a comprehensive catalog of SVs, we performed long‐read sequencing on 16 individuals from six distinct locations: Cap‐Rouge, Portneuf, Baie‐Saint‐Paul, La Pocatière, Bergeronnes, and Cap‐Chat (two individuals by sites, for a total of four individuals from each estuary zone). DNA extraction for oxford nanopore sequencing was performed as described in Gastineau et al. (2023). Large fragments were enriched using the Short Read Eliminator (SRE) XS kit following the manufacturer's instructions (PacBio, Menlo Park, CA, USA). Oxford nanopore library preparations were performed with 1.5 μg of SRE XS enriched genomic DNA using an SQK‐LSK109 ligation sequencing kit following manufacturer's instructions (Oxford Nanopore Technologies, Oxford, UK). Sequencing was performed on MinIon R9.4.1 flow cells on a GridIon instrument (ONT, Oxford, UK) at the Plateforme d'Analyse Génomique at the Institut de Biologie Intégrative et des Systèmes, as part of a research and development project.

### 2.4 Pre‐Processing and SNPs Filtration

To process Illumina sequencing data, we used a custom whole‐genome sequencing pipeline available at (https://github.com/FlorentSylvestre/wgs_sample_preparation). We aligned samples to the most recent version of the three‐spined stickleback reference genome (Nath et al. 2021). To mitigate alignment errors arising from genetic disparities between sexes, we additionally included the Y chromosome reference (Peichel et al. 2020) in males, although omitting the pseudo‐autosomal region (the first 0.34 Mb), as it is already included in the chrXIX reference (X chromosome).

We used BWA‐MEM 0.7.17 (Li and Durbin 2009) to align reads on the reference genome, and the resulting bam files were sorted using samtools v1.8 (Danecek et al. 2011). Subsequent steps involved the removal of PCR duplicates with Picard Toolkit (https://broadinstitute.github.io/picard/), clipping of overlapping reads with bamUtil (https://github.com/statgen/bamUtil) and local realignment of reads around putative indels using GATK 4.1 (Poplin et al. 2018), resulting in an average coverage of 11.2× across our dataset.

To standardize the dataset, we calculated a coverage interval equal to twice the standard deviation around the mean coverage. Samples with a coverage exceeding 17× were subsampled to 17× using samtools view, and those with a coverage below 5× were excluded from subsequent analyses. Additionally, related individuals were removed to mitigate confounding effects on the population structure analyses, based on the phi coefficient of relatedness calculated with the “—relatedness2” argument in vcftools v.0.1.16 (Danecek et al. 2011). We then used the plinkQC R (Meyer 2020) package to retain one individual per related group based on a relatedness threshold value of 0.1.

SNP calling was executed using bcftools v.1.12 Mpileup on a chromosome‐by‐chromosome basis. Sex chromosomes (chrXIX and chrY) and contigs that were not assigned to chromosomes (chrUn) were excluded. We then used bcftools to filter out. Subsequent SNP filtering criteria included the removal of SNPs with more than two alleles and retaining SNPs with a minor allele frequency (MAF) > 0.05, a total coverage between under 4 and above 35, and a genotyping success rate exceeding 50%. SNP filtering was performed using bcftools.

### 2.5 Construction of a Structural Variants Catalog

We combined long‐read sequencing data with the Illumina short‐read sequencing data using a pipeline developed by Lecomte et al. (2024) to find polymorphic indels, inversions, and duplications. Nanopore reads were mapped to the reference genome using Winnowmap 2.03 (Jain et al. 2020, 2022), and reads shorter than 1000 bp were filtered using NanoFilt 2.0.8 (De Coster et al. 2018). SVs from long‐read data were identified using three different callers: Sniffles (Sedlazeck et al. 2018), SVIM (Heller and Vingron 2019), and NanoVar (Cretu Stancu et al. 2017). For the short read data, SVs were identified using Delly (Rausch et al. 2012), Manta (Chen et al. 2016), and Smoove (Pedersen et al. 2020). To ensure better confidence in our SV calls, we created a filtered VCF file that included only SVs detected by at least two callers from each dataset (long or short read). Finally, we combined the SVs identified from both long‐read and short‐read datasets into a single comprehensive dataset. This combined dataset was used for genotyping SVs across all samples using a genome graph approach with VG Giraffe (Sirén et al. 2021). Insertions were primarily identified using long‐read data, while inversions were detected using short‐read data.

### 2.6 Population Structure Analysis

Before performing population structure analysis, we pruned our SNP dataset for linkage disequilibrium (LD) using PLINK v.1.07 (Chang et al. 2015). We applied a conservative *R* ^2^ threshold of 0.5 in windows of 1 kb with a step of 100 bp. The window size corresponds to the LD decay previously calculated in three‐spined sticklebacks (Roesti et al. 2015). This step prevents the estimates of population structure from being driven by a few linked SNPs.

To describe population structure, we utilized both the LD‐pruned SNP and SV datasets across multiple analyses. A principal component analysis (PCA) was performed using the “‐‐pca” argument in PLINK v.1.07. We estimated pairwise genetic differentiation (*F*_ST_) between sampling sites and between estuary zones, using genome‐wide Weir and Cockerham's weighted *F*_ST_. *F*_ST_ values were averaged within 10 kb windows using pixy (Korunes and Samuk 2021) for SNPs and with the “‐‐weir‐fst‐pop” argument in vcftools for SVs. An individual ancestry analysis was also conducted with ADMIXTURE v.1.3.0, testing 2 to 12 possible *K* values from 2 to 12 (Alexander et al. 2009).

We assessed isolation‐by‐distance (IBD) by testing the correlation between pairwise *F*_ST_ values and the logarithm of the Euclidean distance between sampling sites. Euclidean distances were calculated based on geographic coordinates using the SoDA package in R (Chambers 2008). In parallel, we examined the influence of environmental factors, specifically salinity, on genetic differentiation. Salinity measures at each sampling location were obtained from Copernicus satellite data (https://scihub.copernicus.eu/). To further investigate the effect of salinity and geographic distance, we constructed four linear mixed‐effect models (LMEMs) using sample origin as a random effect to account for potential sampling biases: (1) a null model, (2) a salinity‐only model, (3) a distance‐only model, and (4) a combined salinity and distance model. Model selection was performed based on the Akaike Information Criterion corrected for small sample sizes (AICc), using the AICcmodavg package in R (Mazerolle and Linden 2019), with the best‐fitting model being identified by the lowest AICc.

To isolate the effects of distance between marine and middle estuary sites, we conducted an additional IBD analysis. Using the same methods as described above, we constructed two linear models: one excluding Baie‐Saint‐Paul and another including all marine and middle estuary sites. Pearson's correlation tests, implemented with the cor.test function in R, were applied to estimate the relationship between genetic differentiation and Euclidean distance in both cases.

### 2.7 Estimation of Genetic Diversity Indices

To assess genetic diversity and understand population differentiation among the three stickleback populations, we estimated three key parameters: the inbreeding coefficient (*F*_IS_), Tajima's *D*, and nucleotide diversity (Pi). Genetic diversity estimates were calculated on the SNP dataset prior to filtering for MAF, ensuring that both variant and invariant sites were retained in the dataset. We also estimated genetic diversity based on SNPs in 20 kb windows surrounding SVs, but the results mirrored those obtained from the SNP dataset alone. Due to concerns about the independence of the SNP and SV data and the potential lack of added value, we decided to limit our genetic diversity estimates to SNPs in this analysis.

Three populations were analyzed: (1) the fluvial sites, (2) Baie‐Saint‐Paul, and (3) the combined marine and middle estuary sites excluding Baie‐Saint‐Paul. To minimize potential biases due to differing sample sizes, 28 individuals were randomly subsampled from each population. The inbreeding coefficient (*F*_IS_) and Tajima's *D* were estimated using vcftools parameters “‐‐het” and “‐‐TajimaD,” respectively. *F*_IS_ values were computed per individual and summarized by population to compare levels of heterozygosity among the three populations. Mean *F*_IS_ values for each population were statistically compared using a *t*‐test in R (R Core Team 2021). We estimated Tajima's *D* using a window size of 10,000 bp. Given the normal distribution of Tajima's *D* estimates (see Section 3), a *t*‐test was used to compare differences in Tajima's *D* between populations. Nucleotide diversity (Pi) was estimated within each population using Pixy v.1.2.10.beta2 with a window size of 10,000 bp. We used a permutation test to statistically compare the distributions of Pi between populations. This non‐parametric approach involved reshuffling population labels across observed values 10,000 times to generate a null distribution, allowing us to assess the significance of the observed difference in means without assuming a specific data distribution.

### 2.8 AFD Estimation and Cline Analysis

To investigate the presence of a transition zone between freshwater and saltwater sites in the estuary, we performed a cline analysis on both SNPs and SVs. Allelic frequencies within estuary zones and within sites were estimated using the “‐‐freq” option in VCFtools. To focus on alleles that are found at higher frequency in fluvial sites, we identified the major allele at each marker within the fluvial estuary. The frequencies of these minor alleles were combined across sites into a single file. For each allele, we calculated allelic frequency differentiation (AFD) between marine and fluvial estuaries. Outliers were defined as markers with an AFD above the 99th percentile of the AFD distribution, determined using the quantile function in R, and were considered to be likely under diversifying selection given the high degree of differentiation. Outliers were filtered to keep only one outlier per 50 kb window, as linked markers would be redundant and computationally heavy. As a neutral baseline for comparison, we randomly selected the same number of putatively neutral loci as the number of outliers (200). Quantitative cline analyses were independently conducted on outlier and neutral loci using the HZAR package in R (Derryberry et al. 2014). For each marker, we tested five cline models with fixed scaling and varying tail configurations (null, both, none, left and right). Each model was fit using the Metropolis‐Hastings Markov chain Monte Carlo (MCMC) algorithm and evaluated based on maximum likelihood. Model parameters (center, width) were estimated for each model, and the best‐fitting model for each marker was selected based on AIC, with a null model included for comparison. To assess the enrichment of specific model categories in the outlier dataset compared to the neutral dataset, we performed a Fisher's exact test on model counts. The most frequently occurring model across SNPs and SVs in each dataset (outliers and neutral) was identified to represent the overall trend.

### 2.9 Estimation of Demographic Parameters

To investigate gene flow between populations, we conducted demographic modeling using fastsimcoal2 v.2.7 (Excoffier et al. 2013). The models were designed to simulate various migration scenarios between populations. Before proceeding with simulations, we applied an additional filtering step to exclude deviant SNPs that could distort site frequency spectra (SFS) using ngsParalog (https://github.com/tplinderoth/ngsParalog) integrated with ANGSD v.0931, following the methods outlined by Dallaire et al. (2023). For computational efficiency, we focused on a subset of 25 individuals randomly sampled from four sites: two fluvial sites (Cap‐Rouge and Levis) and two marine sites (Betsiamites and Rimouski). Site allele frequencies (SAF) were calculated for each population by chromosome using ANGSD. SNPs were retained if they had a minimum read depth of 4× and were present in at least 90% of the individuals.

Due to the computational demands of demographic modeling and the large‐scale datasets involved, we restricted our analysis to SNPs, as efficient analysis pipelines are readily available for these markers. Although SVs could offer valuable additional insights, the methods for their analysis are computationally intensive at this scale, and the mutation model implemented in *fastsimcoal* is not fully suited to SVs. Next, we used winsfs (https://github.com/malthesr/winsfs) to combine the SAF between pairs of populations and generate the 2D joint SFS. These 2D‐SFS computed for each chromosome were combined to form a whole‐genome 2D‐SFS, which was then converted into the required file format for input into fastsimcoal2.

**FIGURE 2:**
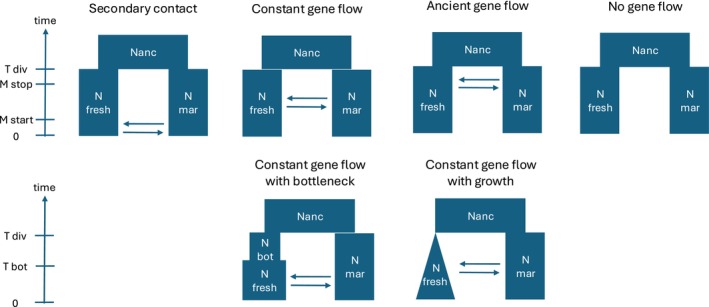
Six demographic models simulating gene flow scenarios between populations of three‐spined sticklebacks in the St. Lawrence Estuary.

We tested six demographic models: (1) no migration since divergence, (2) constant migration since divergence, (3) constant migration with a bottleneck event in the freshwater population, (4) constant migration with population growth after divergence, (5) ancient migration followed by isolation, and (6) secondary contact after isolation (Figure 2). Each model was run 100 times per population pair, with the best run selected based on the highest likelihood of reproducing the observed SFS. Model selection was based on the AIC, following the framework of Meier et al. (2017). To evaluate the robustness of our parameter estimates (Appendix 1), we employed a bootstrapping approach on one population pair (Levis—Betsiamites) due to the redundant results obtained for model selection. We generated 50 bootstrap replicates by subsampling the SFS, removing five chromosomes at a time using custom R scripts. For each replicate, simulations were rerun using the previously identified best‐fitting model identified earlier, and parameter estimates were derived from the distribution across the 50 bootstrap replicates. The best parameter estimates were selected based on their delta likelihood value.

To evaluate the impact of standing genetic variation that is typically under selection in freshwater three‐spined stickleback populations, we repeated the model selection process separately for two chromosomes: chromosome IV, which is strongly associated with freshwater adaptation, and chromosome XV, which contains no known adaptive loci (Roberts Kingman et al. 2021).

## 3 Results

### 3.1 Population Structure Follows a Salinity Gradient Along the Estuary

**FIGURE 3:**
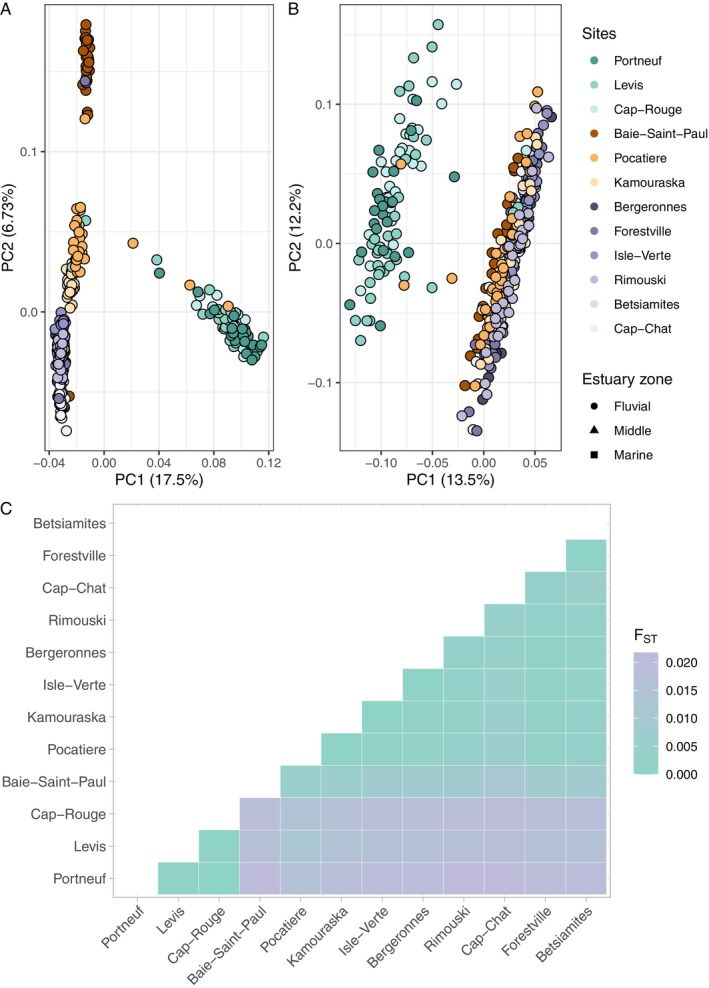
Genetic differentiation among three‐spined stickleback populations across the St. Lawrence Estuary revealed by principal component analysis (PCA) and pairwise *F*_ST_ heatmap: (A) PCA based on SNPs and (B) SVs show distinct genetic structuring, with the first principal component (PC) axis delineating fluvial and saline water populations. SNP‐based PCA further separates middle and marine estuary sites along the second PC axis. (C) Pairwise *F*_ST_ heatmap illustrates genetic differentiation, with higher *F*_ST_ values observed between fluvial and marine estuary sites.

In total, we identified 2,332,752 high‐quality SNPs and 43,737 SVs. For both SVs and SNPs, the first principal component (PC) axis revealed a distinct population structure among anadromous three‐spined sticklebacks inhabiting the Saint‐Lawrence Estuary, delineating two main populations: a freshwater population corresponding to the fluvial estuary and a saline water population encompassing both the middle and marine estuary zones (Figure 3A,B). Additionally, some individuals from the saltwater population cluster with the freshwater population, while some individuals from the freshwater population cluster with the saline water population, suggesting ongoing gene flow between these groups.

When analyzing SNPs, the second PC axis further separated sites from the middle estuary into two clusters, with Baie‐Saint‐Paul on one side and Pocatière and Kamouraska on the other side. This pattern was not observed with SVs. Pairwise *F*_ST_ values further support the presence of these two populations, exhibiting a higher level of genetic differentiation between fluvial and marine sites (*F*_ST fluvial—marine_ = 0.018) compared to other inter‐site pairs (*F*_ST within‐fluvial_ = 0.0005 and *F*_ST within‐middle/marine_ = 0.0025) for both markers (Figure 3C). Notably, Baie‐Saint‐Paul exhibits an average *F*_ST_ of 0.0075 when compared to marine sites, a level of differentiation ~0.005 higher than that observed within the middle and marine group.

**FIGURE 4:**
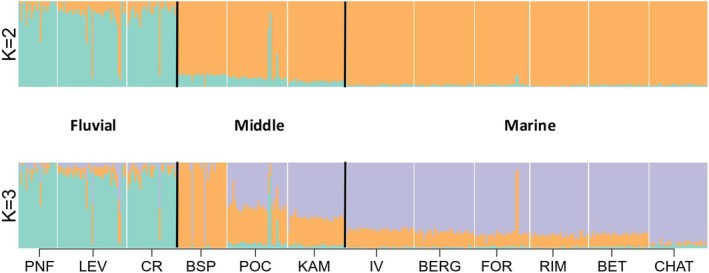
ADMIXTURE plot showing genetic clustering of three‐spined sticklebacks in the St. Lawrence Estuary. At *k* = 2, fluvial and saline water populations (middle and marine) cluster separately, while Baie‐Saint‐Paul forms a distinct third cluster. At *k* = 3, based on cross‐validation error, the optimal *k* value is determined to be 2.

ADMIXTURE analysis revealed a similar pattern, with fluvial and saline water (middle and marine) sites clustering separately at *K* = 2, while Baie‐Saint‐Paul emerged as a third cluster at *K* = 3 (Figure 4). Based on the cross‐validation error, the optimal *K* value was determined to be *K* = 2 (CV‐error = 0.50227).

**FIGURE 5:**
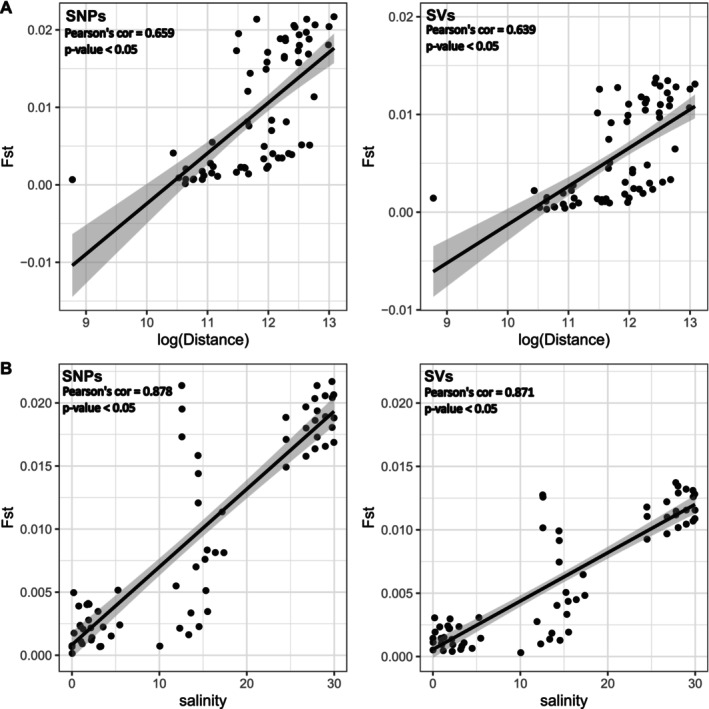
Correlation of genetic differentiation (*F*_ST_) in three‐spined sticklebacks across the St. Lawrence Estuary: (A) Correlation with log‐transformed geographic distance and (B) correlation with salinity, showing a stronger fit than with distance, though the relationship weakens at intermediate salinity levels. For both panels, correlations are shown for SNPs (left) and structural variants (SVs, right). The analysis suggests that salinity provides a stronger explanatory variable for population structure than distance, particularly for SNPs.

**FIGURE 6:**
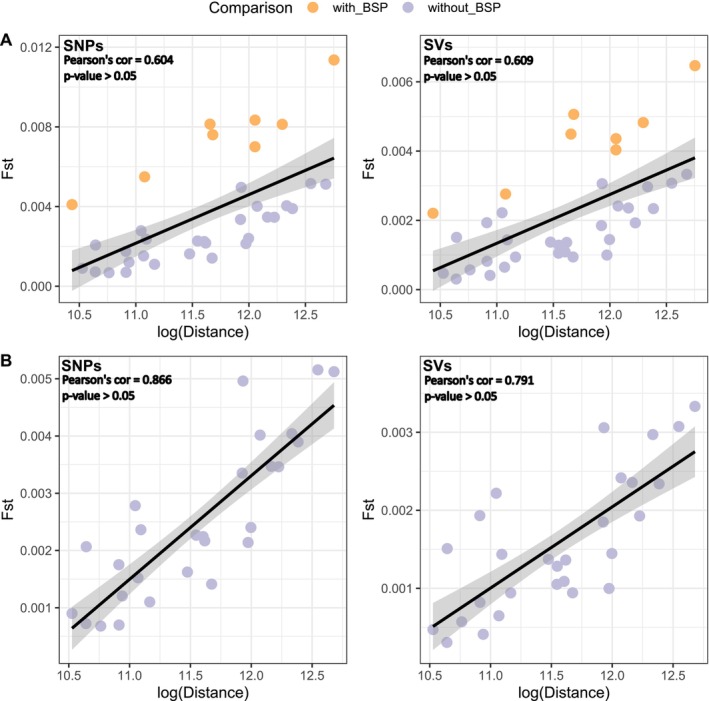
Isolation‐by‐distance (IBD) analysis for SNPs and structural variants (SVs) in the saline water population of three‐spined sticklebacks (A, B). Correlation between genetic differentiation (*F*_ST_) and geographic distance, with and without the inclusion of Baie‐Saint‐Paul (BSP) (A). Excluding BSP increases the correlation strength, suggesting its role as a distinct population within the estuary (B). For SNPs, Pearson's correlation coefficient = 0.6322 (*p* = 2.58e‐09) in panel A and 0.8664 (*p* < 2.2e‐16) in panel B. For SVs, Pearson's correlation coefficient = 0.6414 (*p* = 1.273e‐09) in panel A and 0.7914 (*p* = 3.895e‐13) in panel B.

While IBD alone was insufficient to fully explain the observed population structure across the entire estuary, model selection identified an optimal model incorporating salinity and distance, with SNPs (Table 1, Figure 5). With SVs, model selection couldn't differentiate between a model based on salinity and distance and a salinity‐only model. However, IBD was strongly supported within the saltwater population (middle estuary and marine sites) for SNPs (Pearson's correlation coefficient = 0.6322, *p* = 2.58e‐09; Figure 6A) and for SVs (Pearson's correlation coefficient = 0.6414, *p* = 1.273e‐09), although excluding Baie‐Saint‐Paul increased the correlation strength substantially (SNPs: Pearson's correlation coefficient = 0.8664, *p* < 2.2e‐16; Figure 6B, SVs: Pearson's correlation coefficient = 0.7914, *p* = 3.895e‐13). This suggests that Baie‐Saint‐Paul may represent a third population within the estuary. If IBD were continuous, removing a locality such as Baie‐Saint‐Paul would not necessarily lead to a stronger correlation between distance and *F*_ST_, as the correlation should maintain a consistent pattern across the spatial range.

**TABLE 1:** AIC values from model selection of population structure in the St. Lawrence Estuary, incorporating salinity, distance, and sample origins as random effects. The optimal model for explaining genetic differentiation includes both salinity and distance. The table was produced using the R package AICcmodavg.

Models	Number of parameters	AICc	Delta AICc	Weighted AICc
Distance + salinity	6	−1113.97	0.00	0.8
Salinity	5	−1111.17	2.80	0.2
Distance	5	−1051.57	62.40	0.0
Null	4	−922.96	191.01	0.0

**FIGURE 7:**
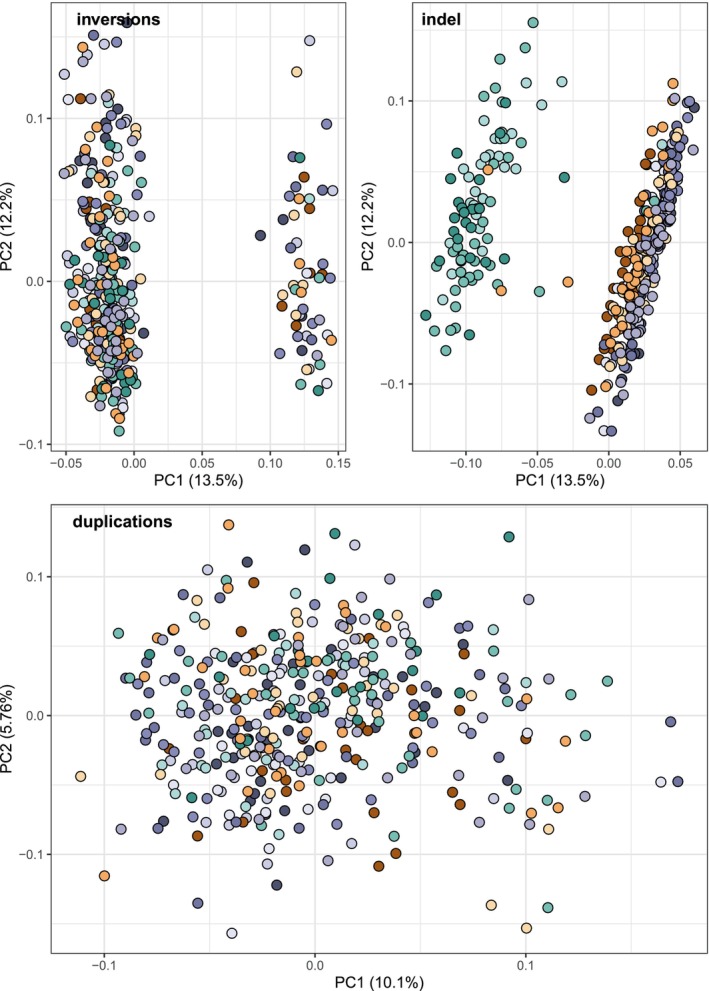
Principal component analysis (PCA) of structural variants (SVs) in three‐spined sticklebacks across the St. Lawrence Estuary: (A) PCA of all insertions and deletions (indels) shows clear differentiation between fluvial and saltwater sites, (B) PCA of inversions reveals two distinct groups, unrelated to estuary zones, with three inversions on chromosomes IX and XI driving the observed structure, and (C) PCA of duplications shows no discernible population structure. Sampling sites are represented by colors, with shapes indicating estuary zones. The analysis highlights distinct patterns of population structure driven by specific SV types.

When considering all SV types, we identified a population structure similar to that found with SNPs, showing a clear delineation between fluvial and saltwater sites (Figure 3B). However, when examining each type of detected SV individually, different patterns emerged. Insertions and deletions (indels) were by far the most abundant types of SVs, with only a small number of inversions (26) and 689 duplications. While indels differentiated between freshwater and saltwater sites, no structure was observed when considering only duplications. Inversions differentiated two groups that were independent from estuary zones (Figure 7) and the first PC axis explained approximately 10% of the total variance. This variance was largely driven by one inversion on chromosome IX.

### 3.2 Genetic Diversity Varies Between Zones of the Estuary

**FIGURE 8:**
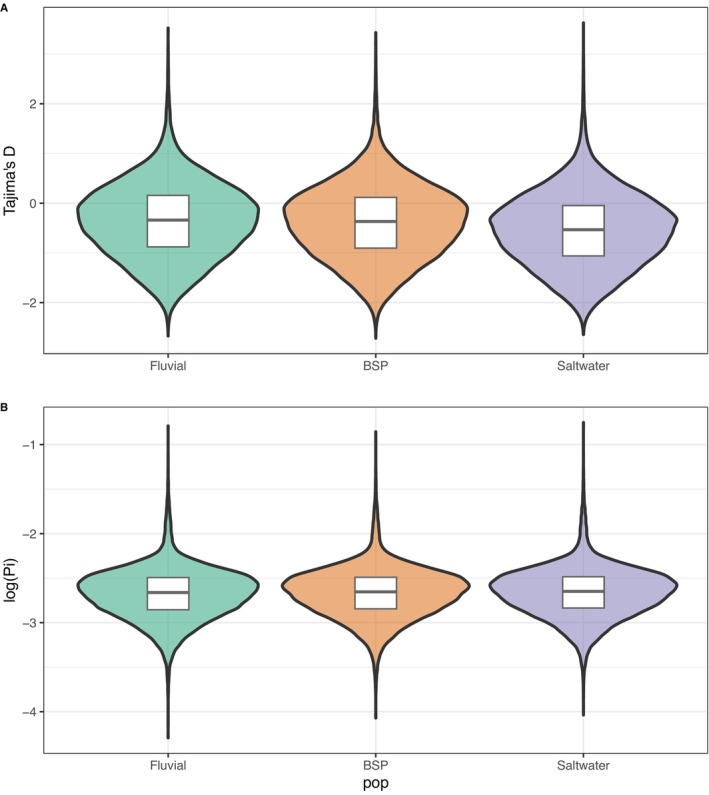
Comparison of nucleotide diversity (Pi) and Tajima's *D* in three‐spined sticklebacks across the St. Lawrence Estuary: (A) Violin plot showing significant differences in Tajima's *D* values among fluvial, Baie‐Saint‐Paul, and saltwater populations. The more negative Tajima's *D* values in the saltwater population suggest stronger purifying selection or recent expansion. (B) Violin plot showing significant differences in nucleotide diversity (Pi) between fluvial and saltwater populations, with lower Pi in the saltwater population (*p* = 5e‐04). In contrast, comparisons involving Baie‐Saint‐Paul showed no significant differences.

Genetic diversity varied significantly between the fluvial and saltwater populations. Tajima's *D* values ranged from −2 to 4 with a slightly negative average across the whole genome, indicating overall neutrality at the whole‐genome scale. Significant differences in average Tajima's *D* were observed between populations (*p* < 2.2e‐16), with the saltwater population showing a more negative value (Tajima's *D* = −0.543) compared to the fluvial population (Tajima's *D* = −0.354) and Baie‐Saint‐Paul (Tajima's *D* = −0.386) (Figure 8A). These more negative values in the saltwater population suggest a recent selective sweep or a population expansion after a bottleneck. However, distinct peaks of highly positive Tajima's *D* were observed on chromosomes IV, VII, and XVI, suggesting regions of balancing selection or population contraction (Appendix 2). No significant differences in the inbreeding coefficient (*F*is) were detected between populations, with all populations exhibiting slightly negative *F*is values (Appendix 2), showing a mild excess of heterozygosity.

Nucleotide diversity was unevenly distributed across the genome, with notable regions of high diversity on chromosomes III and XI (Appendix 2). The average Pi was significantly different between the fluvial and saltwater populations (*p* = 5e‐04, Figure 8B). In both comparisons involving Baie‐Saint‐Paul, there was no significant difference in Pi (*p* > 0.05).

### 3.3 A Cline in Allelic Frequency Along the Estuary Suggests the Presence of Gene Flow and Selection

A clinal variation in allele frequencies was detected along the estuary for both SNPs and SVs, which were identified as AFD‐based outliers. After filtering, we identified 209 outlier SNPs with an AFD above 0.209 (99th percentile of the AFD distribution) and 44 outlier SVs with an AFD above 0.203.

Compared to neutral loci, the outlier SNPs showed significant enrichment for clinal variation models (*p* < 2.2e‐16), indicating that clinal patterns were more common among outlier loci than among neutral loci. Similarly, outlier SVs also showed a significant enrichment for clinal variation models (*p* = 5.54e‐16), supporting a shared pattern of allele frequency shifts across variant types. Among the 209 outlier SNPs, 195 displayed clinal variation, with 178 following a “left” model, where allele frequencies shifted from low values in the marine and middle estuary to high values in the fluvial estuary. Of the remaining SNPs, 14 followed a “both” model, and three followed a “none” model. Among the 44 outlier SVs, 20 followed a “left” model, five followed a “both” model, and one followed a “none” model. Specifically, the null model–indicating no cline–predominated among neutral loci, while 14 outlier SNPs and 18 outlier SVs did not exhibit clinal variation.

The center of the cline was highly consistent between variant types, occurring around Isle‐Verte (225 km for SNPs and 228.5 km for SVs), while the width of the cline was estimated at 196 km for both. Given that the distance was measured from Portneuf, this suggests that the variation extends from the area between Cap‐Rouge and Baie‐Saint‐Paul (approximately 80 km) to the beginning of the marine estuary near Rimouski (around 275 km). The observed cline is shallow with a wide width and no steep decrease in allelic frequency (Appendix 3). Such a cline could be indicative of a combined effect of natural selection and gene flow, with an allele frequency shift arising due to local adaptation but attenuated by ongoing gene flow. The observed pattern could also be a result of historical processes, such as secondary contact between previously isolated populations or differences in migratory behavior between populations.

### 3.4 Asymmetric and Weak Gene Flow Between Populations

**FIGURE 9:**
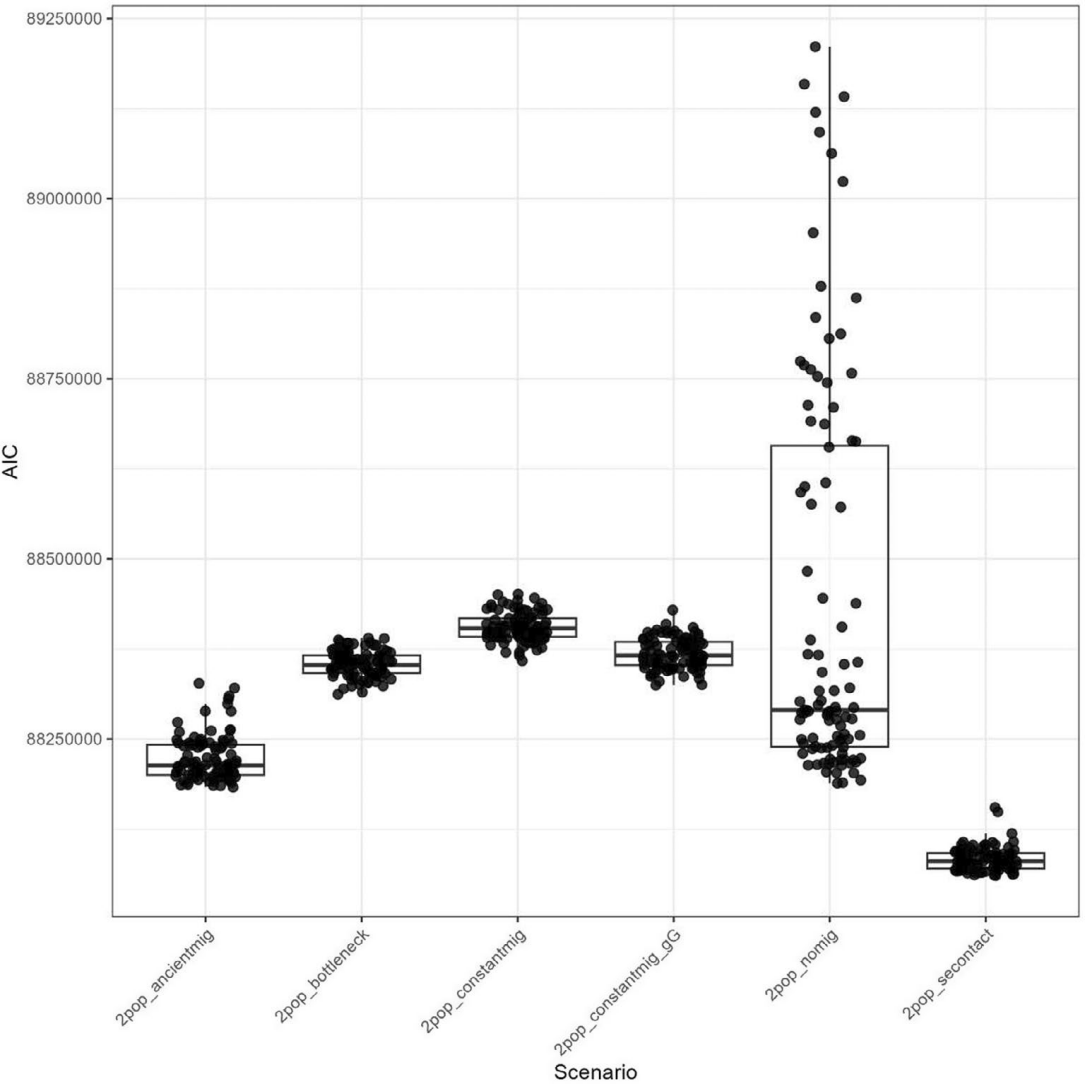
Boxplot of AIC values for six demographic scenarios inferred from fastsimcoal2. The plot compares the AIC distributions of constant migration, constant migration with a bottleneck, constant migration with population growth post‐divergence, ancient migration, secondary contact, and no migration. The secondary contact scenario presents the lowest AIC value.

The secondary contact scenario was consistently supported by model selection based on the best runs produced by fastsimcoal2 across all population pairs for the whole‐genome dataset and for both chromosomes IV and XV. To select a model, we examined the distribution of AIC values across all runs for each scenario (Figure 9). We observed that the secondary contact model displayed a narrow range of AIC values, with a lower mean AIC across runs than other scenarios. Moreover, this model was consistently found to present the run with the lowest AIC value in each comparison; therefore, we selected this model of secondary contact for subsequent bootstrapping analyses of parameter estimates. The parameters of interest for this model included the effective population sizes (Ne) of both fluvial (Nfresh) and saltwater (Nmar) populations, as well as the migration rates between them in both directions (Mig21 and Mig12), their timing of divergence (Tdiv), the timing of the secondary contact (Tmig_stop), and the ancient population size (Nanc).

**FIGURE 10:**
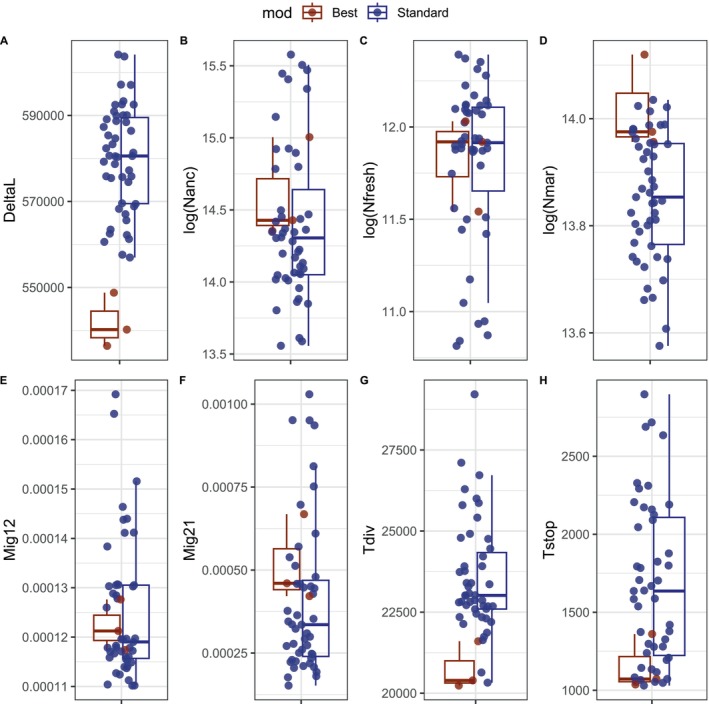
Distribution of demographic parameter estimates for the secondary contact model, visualized as boxplots for eight key parameters (A–H). (A) Delta likelihood (deltaL) across runs. (B) Effective ancestral population size (log‐transformed). (C) Effective population size for fluvial populations (log‐transformed). (D) Effective population size for marine populations (log‐transformed). (E) Migration rate from marine to fluvial populations. (F) Migration rate from fluvial to marine populations. (G) Time of divergence between populations. (H) Timing of secondary contact. Colors distinguish the best runs with the lowest delta likelihood from standard runs.

Three runs presented a substantially lower delta likelihood (mean deltaL = 540,000) than the standard runs (mean deltaL = 579,000) and were selected as the best runs for parameter estimation (Figure 10). For the fluvial population, the mean Ne was 140,312, while for the saltwater population, it was 1,226,787. These values align with expectations, as large Ne values are typical for marine populations (Olsson et al. 2019), while smaller estimates for freshwater populations are consistent with the history of post‐glacial colonization. The migration rate from fluvial to saltwater populations was estimated at 0.0005, while the migration rate in the opposite direction (saltwater to fluvial) was estimated at 0.0001, suggesting that migration is higher in the direction of fluvial to saltwater. The estimated ancestral population size was 2,282,077 individuals and the divergence time was at 20,744 generations. The timing of secondary contact was estimated to be quite recent at 1156 generations.

## 4 Discussion

In this study, we aimed to assess the fine‐scale population structure of three‐spined sticklebacks in the St. Lawrence Estuary using different types of molecular markers to characterize the population genetic structure in this system and evaluate the influence of environmental (salinity) and demographic parameters on observed patterns of population structure. Our results highlight a complex population structure involving three populations that are partially connected via gene flow: a fluvial population, a population at Baie‐Saint‐Paul, and a marine population, with population structure associated with a salinity gradient.

### 4.1 Influence of the Environment on the Population Structure in the St. Lawrence Estuary

The population structure of anadromous three‐spined sticklebacks in the St. Lawrence Estuary detected in this study highlights the complexity of this system. We identified three distinct genetic clusters of three‐spined sticklebacks in the estuary. In our case, differentiation between the fluvial and marine clusters was more pronounced than previously observed, with *F*_ST_≈0.01 at the whole‐genome level and elevated *F*_ST_ values in pairwise comparisons involving Baie‐Saint‐Paul. We also found that considering salinity explained patterns of genetic structure across the estuary better than distance alone, a pattern also reported by McCairns and Bernatchez (2008) using 10 microsatellites (*F*_ST_ = 0.005). Given the well‐documented influence of salinity on fish physiology, population differentiation at opposite extremes of the estuary is expected, even in the absence of physical barriers to gene flow.

While salinity is an important factor influencing fish physiology and ecology by altering osmotic and ionic regulation (Kültz 2015) and modifying trophic interactions (Brucet et al. 2012), it is only one of many selective pressures acting on stickleback populations. Freshwater adaptation is shaped by a broader set of abiotic and biotic factors, including predation, resource availability, and pH, all of which contribute to morphological, physiological, and behavioral differences between freshwater and saltwater three‐spined stickleback populations (Zanella et al. 2015; Smith et al. 2020). However, our study was limited to salinity due to the lack of available data for other relevant environmental variables in the Estuary. Although we used salinity as a proxy for the freshwater environment, the observed population structure is likely influenced by multiple factors characteristic of the fluvial Estuary, rather than salinity alone.

Another plausible explanation for the observed population structure is the coexistence of multiple migratory forms in the Estuary. While St. Lawrence sticklebacks are believed to be anadromous due to the absence of morphological differences and reproductive barriers and the low level of genetic differentiation between populations, it is possible that a more complex pattern of migratory behavior exists in the Estuary. As documented in Japanese stickleback populations (Arai et al. 2020), the freshwater and saltwater populations could be partially anadromous, with some individuals being respectively freshwater and estuarine residents and others being anadromous, leading to two genetically distinct but partially connected populations.

### 4.2 Fine‐Scale Genetic Differentiation at Baie‐Saint‐Paul

The weak fit of the IBD model for comparisons involving Baie‐Saint‐Paul as well as the ancestry coefficients from ADMIXTURE and the separation of Baie‐Saint‐Paul on the second PC axis all support the presence of a third genetic cluster comprising individuals from Baie‐Saint‐Paul. Baie‐Saint‐Paul presents unique environmental conditions, including variable salinity and temperature, which likely impose distinct selective pressures (Couillard et al. 2011). These pressures may contribute to fine‐scale differentiation as seen in other systems, where population divergence occurs over short distances in transitional zones. For instance, Bal et al. (2021) demonstrated genetic differentiation between freshwater and brackish‐water stickleback populations in a short‐range transition zone between Belgium and Netherlands. Similar patterns have been observed in Baltic Sea fish species (Bradbury et al. 2010; Lamichhaney et al. 2012; Guo et al. 2015), where environmental gradients drive population structure despite continuous gene flow. The genetic structure observed at Baie‐Saint‐Paul in this study may reflect such a transition zone.

However, cline analysis positioned Baie‐Saint‐Paul at the extreme left of the cline, far from the cline center at Isle‐Verte, indicating that alleles differentiating fluvial and saltwater populations are not at intermediate frequencies in Baie‐Saint‐Paul. Thus, Baie‐Saint‐Paul appears to be more closely aligned with the fluvial population when considering these loci, and the loci driving differentiation between Baie‐Saint‐Paul and saltwater populations are different from those distinguishing the fluvial and saltwater populations. Consequently, Baie‐Saint‐Paul may constitute a genetically unique population rather than an intermediate group between fluvial and saltwater populations.

As for the saltwater—freshwater population pair, the uniqueness of the Baie‐Saint‐Paul population could also stem from an alternative migratory form. Individuals from this population may be either strictly brackish‐water residents or completely anadromous. Further research, including additional sampling between Cap‐Rouge and Baie‐Saint‐Paul and investigations of morphological and physiological differences, is required to elucidate the drivers of observed patterns of genetic structure.

### 4.3 SV Types Identify Different Patterns of Population Structure

When we looked at each SV type individually, we found that duplications and inversions revealed a different population structure compared to insertions, deletions, and SNPs. The lack of population structure observed when considering duplications can be attributed to their biology and some technical aspects. Duplications are mutations where one or more copies of a DNA segment are produced. They can be bi‐allelic, with only a duplicated and a non‐duplicated state, or multi‐allelic, with a size polymorphism (Hurles 2004). As with microsatellites, multi‐allelic duplications should be more powerful in identifying population structure than bi‐allelic duplications (Narum et al. 2008). However, multi‐allelic duplications are removed by our filters, potentially losing most of the information that duplications can provide in explaining population structure. Moreover, different software treats duplications differently, leading to potential mismatches when merging results. As a result, the small number of duplications that have passed our filtering steps and the inability to identify informative multi‐allelic duplications variants from our catalog could explain the absence of population structure when considering only this type of SV.

With inversions, we found two groups that do not correspond to any tested geographic or phenotypic differences between individuals, with both groups containing individuals from each population. Three large inversions of several mega bases are known to be involved in three‐spined stickleback adaptation to freshwater (Jones et al. 2012). These inversions are not present in their total length in our catalog, as the largest inversion found in our dataset is 5405 bp long. This could be due to the difficulty of the pipeline in identifying large SVs (Lecomte et al. 2024). Since long reads are not paired, they are less efficient in detecting inversions compared to paired short reads. Thus, if an inversion is larger than a long read and too large to be confidently identified by short reads, it would be excluded. This does not imply these inversions are absent but suggests that their detection may be limited by truncation in our dataset. Indeed, when we looked at PCA loadings, one inversion on chromosome IX was found to drive the variation along the first PC axis. This inversion has also been identified as segregating in stickleback populations from the St. Lawrence Estuary by Sylvestre et al. (2023). Contrary to expectations, these inversions did not differentiate freshwater and saltwater sticklebacks, as observed for other structural variants like indels. While we did not aim to characterize in detail the potential role of inversions in local adaptation across an environmental gradient, the unexpected lack of association between known inversions and salinity presents an intriguing pattern that lies beyond the scope of this work. Future studies should further investigate the potential factors driving this observation and further characterize these inversions in relation to ecological and evolutionary processes.

Indels revealed a distinct pattern of population structure that closely mirrored results obtained with SNPs, providing confidence in our observations. Indels are a common type of SV where short sequences of DNA are either inserted or deleted within a genome. These variants can have substantial impacts on gene expression, protein function, and regulatory elements by creating frameshifts in regulatory or coding regions (Massouras et al. 2012; Lin et al. 2017). Because of this, indels can accumulate in a population‐specific manner. Indels have been shown to be powerful markers for detecting population structure across various species (Maw et al. 2015; Mérot et al. 2023; Zhao et al. 2024). For instance, in the Atlantic Salmon, both SNPs and indels identified consistent population structures that correspond to philopatric behavior (Lecomte et al. 2024).

While SVs are a powerful tool for assessing population structure (Conrad and Hurles 2007; Weissensteiner et al. 2020), caution should be exercised when considering only one type of SV. Even if inversions have often been identified in regions driving population structure (Oneal et al. 2014; Tepolt and Palumbi 2020; Vangestel et al. 2024), it does not mean that using all detected inversions without prior knowledge will be an efficient way of studying population structure, as shown by our analysis. Additionally, bi‐allelic duplications detected by our pipeline are not useful for studying population structure. This result corroborates the already frequent practice of removing them from datasets due to their potential to cause false signals.

### 4.4 Gene Flow Shapes the Demographic History of Three‐Spined Sticklebacks in the Estuary

The best‐fitting scenario to explain the demographic history of three‐spined stickleback in St. Lawrence involves a secondary contact between populations with contemporary gene flow. Gene flow plays a fundamental role in shaping the genomic architecture and evolutionary trajectory of populations and species. The strength, direction, and temporal variation of gene flow are critical factors in understanding patterns of genetic differentiation observed in genomic datasets (Tigano and Friesen 2016). The strength and the direction of gene flow directly influence the level of differentiation between populations. When gene flow is strong, populations tend to become more genetically homogenized, counteracting the differentiating effects of genetic drift and local adaptation (Raeymaekers et al. 2014; Bachmann et al. 2020). Due to temporal variation in gene flow, contemporary patterns of diversity and divergence may be attributed to historical demographic events such as founder effects or secondary contact (Santos et al. 2012; Bourgeois et al. 2019; Leder et al. 2021).

In one study, Fang et al. (2018) reconstructed the global phylogeny of three‐spined sticklebacks and identified the eastern Pacific lineage as the oldest, which subsequently colonized the Atlantic via the Bering Seaway and the Arctic Ocean. Based on just two Canadian populations—a freshwater (lake) population from Halifax, Nova Scotia, and a marine population from the southern coast of the St. Lawrence Estuary—they inferred a single colonization event of the western Atlantic by sticklebacks from northern Europe. This event likely followed the deglaciation of the St. Lawrence region, approximately 10,000 to 12,000 years ago (Haines 2023). However, other studies on St. Lawrence fish have shown a pattern of secondary contact between the Atlantic lineage, as discussed above, and an Acadian lineage that colonized the estuary from the south (Bernatchez 1997; Brunner et al. 2001; Dodson et al. 2015). For instance, Bernatchez (1997) found that two distinct rainbow smelled populations co‐exist in the St. Lawrence Estuary, differing primarily in their ancestry: the Acadian lineage occupies the estuary's north coast and ranges from the Great Lakes to the Saguenay, while the Atlantic lineage is present along the south coast, extending to the Gulf and the Maritimes. These findings align with the phylogeny proposed by Fang et al. (2018), who studied populations derived solely from the Atlantic lineage. In our study, we observed a potential signal of secondary contact, suggesting that sticklebacks from the fluvial population may have Acadian origins, while those from the marine population appear to be of Atlantic lineage. This secondary contact hypothesis could explain why the time of divergence between the fluvial and the saltwater populations precedes the deglaciation of the St. Lawrence Estuary (20,000 vs. 12,000 years ago). This pattern could also explain why Baie‐Saint‐Paul forms a distinct genetic cluster, as it may represent the only saltwater site originating from the Acadian lineage.

Post‐glaciation colonization events, such as those in the St. Lawrence Estuary, are typically characterized by bottlenecks followed by population expansions (Nykänen et al. 2019; von Cräutlein et al. 2019). This dynamic likely shaped the demographic history of the three‐spined stickleback, as suggested by the negative mean Tajima's *D* values, which are characteristic of population expansion. Because of the history of colonization, the Acadian lineage may have undergone a stronger bottleneck than the Atlantic lineage, which could explain why we observed a smaller population size for the fluvial population (140,312) compared to the saltwater population (1,226,787).

While moderate and symmetric migration between populations was anticipated, the observed low and asymmetric migration, favoring movement from fluvial to saltwater populations, is plausible. If populations are only partially anadromous, as previously suggested, the overall migration rate would be lower than in a scenario of complete anadromy. Variation in the proportion of anadromous individuals among populations, particularly if this proportion is low, could result in reduced and asymmetric gene flow. Consistent with this hypothesis, McCairns and Bernatchez (2010) demonstrated through common garden experiments that salinity plays a significant role in larval survival in the St. Lawrence Estuary. In their study, freshwater‐origin larvae exhibited no significant changes in survival when reared in different salinities, whereas saltwater‐origin larvae showed decreased survival when reared in freshwater. This pattern suggests that anadromous individuals may be more prevalent in the fluvial estuary than in the marine estuary.

## 5 Conclusion

In this study, we reassessed the population genetic structure of three‐spined sticklebacks in the St. Lawrence Estuary using different types of molecular markers. Our findings revealed a significant differentiation between estuary zones characterized by a clear distinction between fluvial and saltwater populations and a third group at Baie‐Saint‐Paul. The detection of this population structure underscores the effectiveness of genomic markers, particularly SNPs and SVs, in studying fine‐scale population dynamics. Our comparison between SNPs and SVs reinforced the potential of SVs in population genomics studies, emphasizing the importance of the type of SV considered. While deletions and insertions proved valuable in identifying population structure, caution must be exercised when interpreting results based on duplications and inversions. Our demographic analysis highlights the complexity of the evolutionary history of three‐spined stickleback populations in the St. Lawrence Estuary, revealing a pattern of persistent gene flow, with asymmetrical migration rates and evidence of historical bottlenecks, which have likely shaped the current genetic structure of these populations.

Future studies should build on these findings by delving deeper into the genetic mechanisms underlying population differentiation, particularly by examining the genomic architecture of local adaptation to environmental factors such as salinity. Exploring the functional significance of both single nucleotide polymorphisms (SNPs) and structural variants (SVs), as well as their interactions with recombination rate variation, will provide crucial insights into the evolutionary processes shaping the genetic landscape of these populations.

Overall, our study emphasizes the intricate interplay between gene flow and environmental heterogeneity in shaping patterns of population structure, offering valuable insights into the broader mechanisms driving genetic differentiation and species evolution in complex ecosystems like the St. Lawrence Estuary.

## Author Contributions

**Sann Delaive:** investigation (lead), methodology (lead), writing – original draft (lead). **Florent Sylvestre:** data curation (supporting), investigation (supporting), methodology (supporting), writing – review and editing (equal). **Amanda Xuereb:** conceptualization (supporting), formal analysis (supporting), investigation (supporting), methodology (supporting), writing – review and editing (equal). **Laurie Lecomte:** data curation (equal), methodology (supporting). **Brian Boyle:** funding acquisition (supporting), methodology (supporting), resources (supporting). **Christian Otis:** methodology (supporting), resources (supporting). **Louis Bernatchez:** conceptualization (lead), funding acquisition (lead), resources (equal), supervision (equal). **Nicolas Derome:** resources (equal), supervision (equal), writing – review and editing (equal).

## Conflicts of Interest

The authors declare no conflicts of interest.

## Data Availability

The dataset generated in this study will be available in DRYAD. Source code for population genomic analysis performed in this paper can be found at GitHub (https://github.com/Sannouche/Population_genomics).

## Appendix 1 Parameter Estimates Used in Fastsimcoal2 Demographic Models to Assess Gene Flow Between Fluvial and Marine Populations of the St. Lawrence Estuary.

**Table:**

Parameters	Type	Range of values
Ancestral size (Nanc)	Unif	0.001 to 10
Marine effective size (Nmar)	Unif	200 to 200,000
Fluvial effective size (Nfresh)	Unif	200 to 200,000
Bottleneck population size (BotPop)	Unif	0.01 to 0.1
Migration from fluvial to marine (Mig21)	Logunif	0.0001 to 0.02
Migration from marine to fluvial (Mig12)	Logunif	0.0001 to 0.02
Growth in freshwater (Gr1)	Unif	−0.001 to 0.001
Time of divergence (Tdiv)	Unif	1000 to 20,000
Time of secondary contact (Tmig_Stop)	Unif	1000 to 20,000
Time of the end of the bottleneck (Tbot_End)	Unif	100 to Tdiv
Time of the end of the growth (Tgr1)	Unif	1000 to Tdiv

## Appendix 2

Average nucleotide diversity (Pi), Tajima's *D*, and inbreeding coefficient (*F*_is_) values for three‐spined stickleback populations and Genomic distribution of Pi and Tajima's *D* across the St. Lawrence Estuary. (A) Table including values for each population: Fluvial Estuary, Baie‐Saint‐Paul, and Saltwater Estuary. No significant differences were observed in *F*_is_ between populations, with all exhibiting slightly negative values, indicating a mild excess of heterozygosity. (B) Manhattan plot of Tajima's *D* across the genome, with peaks on chromosomes IV, VII, and XVI indicating possible balancing selection or population contraction. (C) Manhattan plot of average Pi across the genome, highlighting regions of high diversity on chromosomes III and XI, illustrating genetic diversity patterns that differ significantly between fluvial and saltwater populations.

## Appendix 3

Allelic Frequency Clines of Outlier and Neutral SNPs Along the St. Lawrence Estuary in Three‐Spined Sticklebacks. This figure illustrates clinal shifts in allelic frequencies for 195 outlier SNPs (orange) and neutral SNPs (gray) as a function of distance from Portneuf, with vertical dashed lines indicating shift between estuary zones.
